# Correction: Interventions facilitating access to perinatal care for migrant women without medical insurance: A scoping review protocol

**DOI:** 10.1371/journal.pone.0274983

**Published:** 2022-09-15

**Authors:** The PLOS ONE Staff

There are errors in the author affiliations. The correct affiliations are as follows:

Drissa Sia^1,2^, Eric Tchouaket Nguemeleu^1,3^, Idrissa Beogo^4,5^, Catherine Séguin^6^, Geneviève Roch^7^, Janet Cleveland^8^, Christina Greenaway^9^

**1** Département des sciences infirmières, Université du Québec en Outaouais, Saint-Jérôme, Québec, Canada, **2** Département de médecine sociale et préventive, École de santé publique, Université de Montréal, Montréal, Québec, Canada, **3** Département de gestion, d’évaluation et de politique de santé, École de santé publique, Université de Montréal, Montréal, Québec, Canada, **4** École des sciences infirmières, | School of Nursing, Faculty of Health Sciences, University of Ottawa, Ottawa, Ontario, Canada, **5** College of Nursing, Rady Faculty of Health Sciences, University of Manitoba, Winnipeg, Manitoba, Canada, **6** Library, Université du Québec en Outaouais, Saint-Jérôme, Québec, Canada, **7** Faculty of Nursing, Université Laval, Québec, Québec, Canada, **8** McGill University Health Centre, McGill University, Montreal, Quebec, Canada, **9** Jewish General Hospital and Department of Medicine, McGill University, Montreal, Quebec, Canada.

The publisher apologizes for the errors.
