# Supplementary material for: Interventions facilitating access to perinatal care for migrant women without medical insurance: A scoping review protocol
Source: PLoS One. 2022 Mar 14;17(3):e0265232. doi: 10.1371/journal.pone.0265232 (PMC8920260; doi:10.1371/journal.pone.0265232)
Supplement: S1 Appendix — (DOCX) [file pone.0265232.s002.docx]

**Appendix 1:** Complete list of countries considered

***Low- and middle-income countries***

| Afghanistan | Guinea-Bissau | Sierra Leone |
| --- | --- | --- |
| Burkina Faso | Haiti | Somalia |
| Burundi | Korea, Dem. People's Rep. | South Sudan |
| Central African Republic | Liberia | Sudan |
| Chad | Madagascar | Syrian Arab Republic |
| Congo, Dem. Rep | Malawi | Tajikistan |
| Eritrea | Mali | Togo |
| Ethiopia | Mozambique | Uganda |
| Gambia, The | Niger | Yemen, Rep. |
| Guinea | Rwanda | Papua New Guinea |
| Angola | Honduras | Philippines |
| Algeria | India | São Tomé and Principe |
| Bangladesh | Kenya | Senegal |
| Benin | Kiribati | Solomon Islands |
| Bhutan | Kyrgyz Republic | Sri Lanka |
| Bolivia | Lao PDR | Tanzania |
| Cabo Verde | Lesotho | Timor-Leste |
| Cambodia | Mauritania | Tunisia |
| Cameroon | Micronesia, Fed. Sts. | Ukraine |
| Comoros | Moldova | Uzbekistan |
| Congo, Rep. | Mongolia | Vanuatu |
| Côte d'Ivoire | Morocco | Vietnam |
| Djibouti | Myanmar | West Bank and Gaza |
| Egypt, Arab Rep. | Nepal | Zambia |
| El Salvador | Nicaragua | Zimbabwe |
| Eswatini | Nigeria | Montenegro |
| Ghana | Pakistan | Namibia |
| Albania | Fiji | North Macedonia |
| American Samoa | Gabon | Paraguay |
| Argentina | Georgia | Peru |
| Armenia | Grenada | Russian Federation |
| Azerbaijan | Guatemala | Samoa |
| Belarus | Guyana | Serbia |
| Belize | Indonesia | South Africa |
| Bosnia and Herzegovina | Iran, Islamic Rep. | St. Lucia |
| Botswana | Iraq | St. Vincent and the Grenadines |
| Brazil | Jamaica | Suriname |
| Bulgaria | Jordan | Thailand |
| China | Kazakhstan | Tonga |
| Colombia | Kosovo | Turkey |
| Costa Rica | Lebanon | Turkmenistan |
| Cuba | Libya | Tuvalu |
| Dominica | Malaysia | Venezuela, RB |
| Dominican Republic | Maldives |  |
| Equatorial Guinea | Marshall Islands |  |
| Ecuador | Mexico |  |

***High-income countries***

| Andorra | Greece | Palau |
| --- | --- | --- |
| Antigua and Barbuda | Greenland | Panama |
| Aruba | Guam | Poland |
| Australia | Hong Kong SAR, China | Portugal |
| Austria | Hungary | Puerto Rico |
| Bahamas, The | Iceland | Qatar |
| Bahrain | Ireland | Romania |
| Barbados | Isle of Man | San Marino |
| Belgium | Israel | Saudi Arabia |
| Bermuda | Italy | Seychelles |
| British Virgin Islands | Japan | Singapore |
| Brunei Darussalam | Korea, Rep. | Sint Maarten (Dutch part) |
| Canada | Kuwait | Slovak Republic |
| Cayman Islands | Latvia | Slovenia |
| Channel Islands | Liechtenstein | Spain |
| Chile | Lithuania | St. Kitts and Nevis |
| Croatia | Luxembourg | St. Martin (French part) |
| Curaçao | Macao SAR, China | Sweden |
| Cyprus | Malta | Switzerland |
| Czech Republic | Mauritius | Taiwan, China |
| Denmark | Monaco | Trinidad and Tobago |
| Estonia | Nauru | Turks and Caicos Islands |
| Faroe Islands | Netherlands | United Arab Emirates |
| Finland | New Caledonia | United Kingdom |
| France | New Zealand | United States |
| French Polynesia | Northern Mariana Islands | Uruguay |
| Germany | Norway | Virgin Islands (U.S.) |
| Gibraltar | Oman |  |

***Source:*** World Bank in 2021^22^
