# Supplementary material for: Interventions facilitating access to perinatal care for migrant women without medical insurance: A scoping review protocol
Source: PLoS One. 2022 Mar 14;17(3):e0265232. doi: 10.1371/journal.pone.0265232 (PMC8920260; doi:10.1371/journal.pone.0265232)
Supplement: S2 Appendix — (DOCX) [file pone.0265232.s003.docx]

**Appendix 2:** Extraction Grid

| Name of authors & year of publication |  |  |
| --- | --- | --- |
| Title |  |  |
| country |  |  |
| abstract |  |  |
| Objective |  |  |
| Methodology | Study design |  |
|  | Study population (age, country of origin) |  |
|  | Sample size |  |
|  | Type of analysis |  |
| Interventions | Antenatal care |  |
|  | Immediate obstetric and neonatal care |  |
|  | Postpartum care |  |
|  | Newborn care |  |
|  | Policies and practices in favor of this care |  |
|  | Findings |  |
|  | Impact |  |
|  | Strengths |  |
|  | Weaknesses |  |
|  | Implementation cost of these interventions |  |
| Limits |  |  |
| Comments |  |  |
