# Supplementary material for: Interventions facilitating access to perinatal care for migrant women without medical insurance: A scoping review protocol
Source: PLoS One. 2022 Mar 14;17(3):e0265232. doi: 10.1371/journal.pone.0265232 (PMC8920260; doi:10.1371/journal.pone.0265232)
Supplement: Attachment — Submitted filename: Response to Reviewers.docx [file pone.0265232.s004.docx]

Kelli K Ryckman

Academic Editor

PLOS ONE

Dear Kelli K Ryckman

We would like to thank the reviewers for their thorough critics and comments concerning our manuscript " Interventions facilitating access to perinatal care for migrant women without medical insurance: a scoping review protocol " (PONE-D-21-27767). The manuscript has been revised according to the journal requirements and to comments of our reviewers and we have addressed all their questions.

Our answers are written in italics under each comment made by the reviewers and the changes related to these comments are presented in the text of the article, with track changes.

***Before answering comments, we state the following:***

***Financial disclosure****:*

*Yes, this project has been funding by the SHERPA University Institute via 2020-2021 annual competition to support researchers of the SHERPA University Institute and by the Social Sciences and Humanities Research Council (SSHRC) via SSHRC institutional grants - winter 2021 competition.*

*The funders had no role in study design, data collection and analysis, decision to publish, or preparation of the manuscript*

***Data available statement:***

*As this is a scoping review protocol, data stemmed from the included papers will be available on the published paper related to this protocol under the form of supplementary files. This includes the concept plan, the flow chart and included articles and their characteristics.*

I. **Journal Requirements:**

When submitting your revision, we need you to address these additional requirements.

1. Please ensure that your manuscript meets PLOS ONE's style requirements, including those for file naming. The PLOS ONE style templates can be found at

<https://journals.plos.org/plosone/s/file?id=wjVg/PLOSOne_formatting_sample_main_body.pdf> and

<https://journals.plos.org/plosone/s/file?id=ba62/PLOSOne_formatting_sample_title_authors_affiliations.pdf>

***Response****: We followed PLOS ONE's style requirements for editing our manuscript*

2. Thank you for stating the following financial disclosure:

“Yes, this project has been funding by the SHERPA University Institute via 2020-2021 annual competition to support researchers of the SHERPA University Institute and by the Social Sciences and Humanities Research Council (SSHRC) via SSHRC institutional grants - winter 2021 competition”

***Response:*** *Yes, this project has been funding by the SHERPA University Institute via 2020-2021 annual competition to support researchers of the SHERPA University Institute and by the Social Sciences and Humanities Research Council (SSHRC) via SSHRC institutional grants - winter 2021 competition*

*We took into account this recommendation. This information will be put in the online system.*

*As recommended, we removed it from the manuscript.*

*Please see on p.8*

Please state what role the funders took in the study.  If the funders had no role, please state: "The funders had no role in study design, data collection and analysis, decision to publish, or preparation of the manuscript."

***Response:*** *It’s done accordingly in the manuscript as follow: The funders had no role in study design, data collection and analysis, decision to publish, or preparation of the manuscript. Please see on p.8*

Please include this amended Role of Funder statement in your cover letter; we will change the online submission form on your behalf.

3. We note that you have stated that you will provide repository information for your data at acceptance. Should your manuscript be accepted for publication, we will hold it until you provide the relevant accession numbers or DOIs necessary to access your data. If you wish to make changes to your Data Availability statement, please describe these changes in your cover letter and we will update your Data Availability statement to reflect the information you provide.

***Response:*** *Data available statement:*

*As this is a scoping review protocol, data stemmed from the included papers will be available on the published paper related to this protocol under the form of supplementary files. This includes the concept plan, the flow chart and included articles and their characteristics.*

*This statement is mentioned in the cover letter and we updated this information in the paper (see p 9)*

4. Thank you for stating the following in the Acknowledgments Section of your manuscript:

“Acknowledgements:

We would like to thank the SHERPA University Institute and The Social Sciences and Humanities Research Council (SSHRC) for funding the project.

***Response:*** *We added this acknowledgment statement in the revised manuscript. Please see on p.8*

Funding:

This project has been funding by the SHERPA University Institute via 2020-2021 annual competition to support researchers of the SHERPA University Institute and by the Social Sciences and Humanities Research Council (SSHRC) via SSHRC institutional grants - winter 2021 competition.”

We note that you have provided additional information within the Acknowledgements Section. Please note that funding information should not appear in the Acknowledgments section or other areas of your manuscript. We will only publish funding information present in the Funding Statement section of the online submission form.

Please remove any funding-related text from the manuscript and let us know how you would like to update your Funding Statement. Currently, your Funding Statement reads as follows:

 “Yes, this project has been funding by the SHERPA University Institute via 2020-2021 annual competition to support researchers of the SHERPA University Institute and by the Social Sciences and Humanities Research Council (SSHRC) via SSHRC institutional grants - winter 2021 competition”

Please include your amended statements within your cover letter; we will change the online submission form on your behalf.

***Response:*** *Thanks for the comment.*

*In the revised manuscript, we removed the funding statement. It is now mentioned in the cover letter. Our amended statement is as follow: Yes, this project has been funding by the SHERPA University Institute via 2020-2021 annual competition to support researchers of the SHERPA University Institute and by the Social Sciences and Humanities Research Council (SSHRC) via SSHRC institutional grants - winter 2021 competition*

5. We note that you have referenced (Open Grey, Wholis, unpublished reports]) which has currently not yet been accepted for publication. Please remove this from your References and amend this to state in the body of your manuscript: (“Open Grey, Wholis, [unpublished reports]”) as detailed online in our guide for authors

<http://journals.plos.org/plosone/s/submission-guidelines#loc-reference-style>

***Response:*** *We merely removed the grey literature (Open Grey, Wholis, unpublished reports).*

6. Please include your full ethics statement in the ‘Methods’ section of your manuscript file. In your statement, please include the full name of the IRB or ethics committee who approved or waived your study, as well as whether or not you obtained informed written or verbal consent. If consent was waived for your study, please include this information in your statement as well.

***Response:*** *An excerpt related to the ethical consideration is now transferred to Methods section, and we added the following statement ‘Informed written or verbal consent is not required. Data will not be retrieved individuals. (See page 7 in the manuscript)*

7. Please include captions for your Supporting Information files at the end of your manuscript, and update any in-text citations to match accordingly. Please see our Supporting Information guidelines for more information: <http://journals.plos.org/plosone/s/supporting-information>.

***Response:*** *The PRISMA-ScR file is updated and appended to the manuscript.*

8. We note that this manuscript is a systematic review or meta-analysis; our author guidelines therefore require that you use PRISMA guidance to help improve reporting quality of this type of study. Please upload copies of the completed PRISMA checklist as Supporting Information with a file name “PRISMA checklist”.

***Response:*** *Our manuscript is a scoping review (protocol), that is why we filled out the PRISMA-ScR checklist.*

II. **Reviewer #1:**

This is a well written scoping review proposal to identify interventions to improve access to perinatal care for migrant women without medical insurance. A clear structure for the review is given, with a comprehensive search strategy, inclusion and exclusion criteria given. I have one main critique: Within the data analysis section it states in step 4 that they will be: “discussing the implications for future research, clinical practice, and health policy.” The methodological framework for scoping reviews developed by Arksey & O’Malley (2005) specifically states that due to the quality of studies not being assessed within a scoping review that as a result the generalisability and robustness of the studies cannot be determined. Careful consideration therefore needs to be given to the extent to which their scoping review results will lead to implications for policy and practice, or further justification given if they do feel this is appropriate.

***Response:*** *Thanks for your comment that is very pertinent. So far, very few is done in terms of review related to the issue of the interventions facilitating the access to perinatal care for migrant women without medical insurance. Therefore, this scoping review is to be considered as a first step of the project. Despite its exploratory feature, in the sixth step (optional) according to Arskey and O’Malley’s framework, we will convey an expert deliberative dialogue, in order to strengthen the potential outcomes.*

All other comments are of a very minor nature: The sentence in the introduction: “These women also have a high prevalence of postpartum depression6, parasitic7 and other infectious diseases (Hepatitis B; Hepatitis C; HIV) / AIDS) due to the challenges experienced in accessing perinatal care.” Currently reads as though women are at risk of infectious diseases due to the challenges of accessing perinatal care, but my understanding is that these infections are already present but make accessing care early all the more necessary.

***Response:*** *Thanks for this comment. For more comprehension, we reframed the excerpt as follows: ‘These women also have a high prevalence of postpartum depression6, parasitic7 and other infectious diseases (Hepatitis B; Hepatitis C; HIV) / AIDS). The fact they experienced challenges in accessing perinatal care8, constitutes a double burden.’*

*Please see on p.3*

No explanation/justification is given as to why the search begins from the date 2000.

***Response:*** *As suggested, explanation justifying why the search begins from the date 2000 is provided in the text as follows: ‘2000 was a landmark turning point for the entire world with the formulation of the eight Millennium Development Goals (MDGs), where emphasis was put in poverty and hunger reduction (MDG 1) the promotion of gender equality and empower of women (MDG 3) and among other child mortality reduction (MDG 4).’ Please see on p.5*

If there are any questions regarding the content of an article, will the article authors be contacted in any way for clarification?

***Response:*** *Yes, for any question regarding the content of an article, the authors will be contacted for further clarification?*

*We mentioned this in the text. Please see on p. 5*

Please ensure correct ordering of Tables / Appendices – ie in the text Table 3 is referred to prior to Table 2 and Appendix 2 prior to Appendix 1 – please reorder correctly.

***Response:*** *We re-ordered the tables / Appendices accordingly.*

*Please see on p. 5 & 5 & 12 to 24*

Please note the abbreviation is incorrect in several places – ie PMWMI has been replaced by the incorrect PMWNI (seen in abstract and introduction – but please check throughout).

***Response:*** *Sorry for the typos. We have checked throughout and made the corrections accordingly. Please see on p.2 & 3*

Table 3: In English “échappées belles" are normally termed “near misses”. I had to Google for a translation to the above term, so it could help some readers if both terms were used within the table.

***Response:*** *We considered both terms within the table (see p. 13)*

Appendix 1: Is it possible to rotate the table by 90 degrees for publication, so that the columns become rows to make it easier for the reader to see what data will be extracted.

***Response:*** *Thanks for the suggestion. We reshaped the tables as suggested.*

*It is easily readable now.*

Appendix 2: Please add the World Bank reference from the text to the Appendix as well
***Response:*** *We now added the World Bank reference to the Appendix.*

Figure 1: In the legend it states “4: Time horizon ...” – however this is not added into the actual diagram at present.

***Response:***

*The legend is now adjusted. The No4 is added, it corresponds to the time horizon of perinatal care.*

III. **Reviewer #2**:

The authors of this scoping review are seeking to addresses an important issue, and their overall approach is well-suited to the topic of inquiry. It will make an important contribution to the literature on maternity care for migrant women, and it is strengthened by the element of expert consultation which is included. However, the protocol is unclear in places and would benefit from further refining. I would suggest the following points are considered:

**Major issues**

1. p.3 – In the opening paragraph, the authors make reference to challenges faced by migrant women accessing maternity care in high-income countries. However, listed in Appendix 2 it is stated that both high-income countries as well as low- and middle-income countries will be included in this review, which is a little confusing for the reader and needs to be clarified. In addition, it will be helpful for the authors somewhere in the paper to discuss how they will deal with the differences in findings from the varying contexts of HICS’s vs LMIC’s.

***Response:*** *Thanks for this pertinent comment. We rephrased first sentence of introduction with references as follow: “Although the phenomenon, known as the ‘healthy immigrant effect’, has been repeatedly reported, in many countries, migrant women face inadequate pregnancy …”.*

*We also added excerpt in the data analysis section, as follows: ‘comparing the interventions implanted and their outcomes in regard to the setting (high-, medium- or low-income country)’ (see p. 6).*

2. p.3 – The introduction paints a picture of catch-all poor perinatal outcomes for migrant women. As the authors correctly acknowledge, migrant women constitute a heterogeneous group and the research is equivocal about obstetric outcomes for some migrant women, citing the healthy migrant effect. A more nuanced introduction would strengthen the introductory section.

***Response: We*** *now nuanced our introduction as follow: “Although the phenomenon, known as the ‘healthy immigrant effect’, has been repeatedly reported, in many countries, migrant women face inadequate pregnancy ….” (see p 3)*

3. p.4 – In the ‘population’ paragraph, the authors quote the definition of ‘migrant’ which describes voluntary migration, but then later on state that studies which include asylum seekers will be considered. Although migrant categories are hard to define and overlapping, for the purpose of this scoping review, it would be helpful to clearly outline what the authors are referring to by ‘migrant women’ – is this all categories of migrants, whether forced or voluntary, as the search terms suggest? If so, the definition of ‘migrant women’ which the authors cite needs adapting. Does it include those who migrate within their country (as the International Organization for Migration definition includes)?

***Response:*** *Thank you for this pertinent comment. In order to adhere to the scope of our population of study, we reshape the excerpt as follow: “a person who voluntarily or forced to move whether within a country or across an international border, temporarily or permanently, and for a variety of reasons, in order to improve his or her material and social conditions, his or her future prospects or those of his or her family” (see p. 4)*

4. p.4 – In the ‘intervention’ paragraph, the authors discuss the intervention in the PICO framework as being the actual maternity or newborn care which women receive. However, on p.3 the research question states that the authors are interested in “interventions that facilitate access to perinatal care”. There needs to be further clarity as to whether it’s perinatal care itself or interventions to facilitate access to perinatal care that is the topic of inquiry, which then needs to be reflected in the protocol title and the intervention.

***Response:*** *Your comment is very pertinent but also* *picky.*

*In this study, we see an intervention as all cares given to mother and\or newborn as well as any policy in that domain. And then, we look at any weakness and strengths regarding these perinatal cares and policies in terms of the final outcome that the accessibility.*

5. p.4 – Newborn care is listed in the ‘intervention’ paragraph but related search terms are not included in the search strategy.

***Response:*** *Thanks for this comment. We now include in the search strategy newborn cares and neonatal care. A quick search in CINHAL yield a difference in the number of references obtained*. (See table 3, p. 14)

6. p.4 – In the ‘comparator and design’ section, the authors state the study inclusion criteria. From my understanding of this scoping review and according to the PICO framework, there is no ‘comparator’ in this review, so this needs to be reworked.

***Response:*** *We agree with the comment. There is no comparator. That is true in our mind. In that section, our emphasis clearly focusses on the design.*

*That is, this sentence was added in the Comparator and Design section: ‘No comparison is foreseen. (See p. 4)*

7. p.4/5 – In the ‘data sources’ paragraph, it is stated that grey literature will be included, but earlier on p.4 in the ‘comparator and design’ section, the authors state that all empirical scientific studies will be included – it would help the reader to understand the inclusion criteria if the authors state whether they are only going to include peer-reviewed published studies or studies that are not peer-reviewed.

***Response:*** *Based on the editorial recommendation, we merely remove the grey literature (Open Grey, Wholis, unpublished reports) from data sources section.*

*Thanks for the comment. (See p. 5)*

**Minor issues**
p.3 – In the second paragraph of the introduction, the authors state that using a scoping review will allow them to synthesise the interventions, and yet there is not further mention of data synthesis in the paper. Arksey and O’Malley (2005) do not require data synthesis in their approach to scoping reviews – this just needs to be clarified in the manuscript.

***Response:*** *Thanks for this comment.*

*Yes, we will adhere to Arkey and O’Malley framework. That is, at its 5^th^ step, we plan to provide a synthesis.*

**PLOS requirements**

While revising your submission, please upload your figure files to the Preflight Analysis and Conversion Engine (PACE) digital diagnostic tool, <https://pacev2.apexcovantage.com/>. PACE helps ensure that figures meet PLOS requirements. To use PACE, you must first register as a user. Registration is free. Then, login and navigate to the UPLOAD tab, where you will find detailed instructions on how to use the tool. If you encounter any issues or have any questions when using PACE, please email PLOS at [figures@plos.org](mailto:figures@plos.org). Please note that Supporting Information files do not need this step.

***Response****: We did so, and our figure 1 meet PLOS requirements*
