# Supplementary material for: Interventions facilitating access to perinatal care for migrant women without medical insurance: A scoping review protocol
Source: PLoS One. 2022 Mar 14;17(3):e0265232. doi: 10.1371/journal.pone.0265232 (PMC8920260; doi:10.1371/journal.pone.0265232)
Supplement: Attachment — Submitted filename: Response to Reviewers-14janv2022.docx [file pone.0265232.s005.docx]

Kelli K Ryckman

Academic Editor

PLOS ONE

Dear Kelli K Ryckman

We would like to thank the reviewers for their thorough critics and comments concerning our manuscript " Interventions facilitating access to perinatal care for migrant women without medical insurance: a scoping review protocol " (PONE-D-21-27767). The manuscript has been revised according to the journal requirements and to comments of our reviewers and we have addressed all their questions.

Our answers are written in italics under each comment made by the reviewers and the changes related to these comments are presented in the text of the article, with track changes.

I. **Journal Requirements:**

Please review your reference list to ensure that it is complete and correct. If you have cited papers that have been retracted, please include the rationale for doing so in the manuscript text, or remove these references and replace them with relevant current references. Any changes to the reference list should be mentioned in the rebuttal letter that accompanies your revised manuscript. If you need to cite a retracted article, indicate the article’s retracted status in the References list and also include a citation and full reference for the retraction notice.

***Response****: Reference list has been revised and no change made.*

II. **Reviewer #1:**

Thank you for the opportunity to re-review this article. There are still a couple of areas that require clarification before publication to ensure consistency throughout the manuscript.

**1.** There still seems to be some confusion over the inclusion of grey literature. The abstract states “Grey literature will also be searched” and the search strategy states that “(iv) sites of non-governmental organizations (Médecin du monde, Médecin sans Frontières (MSF), United Nations High Commissioner for Refugees (UNHCR)” will be searched, which would be expected to find grey literature. However, the response to the reviewers states that grey literature has been removed from the data sources. Please ensure the whole manuscript is consistent regarding this, making it explicit whether unpublished studies will be included.

***Response****: Thanks for the comment.* *We will be considering only publications from the three main non-governmental organizations involved in addressing issues concerning migrant women without medical insurance, namely Médecin du monde, Médecin sans Frontières (MSF), United Nations High Commissioner for Refugees (UNHCR). There for we will not search into any other sources except the aforementioned.*

*Through out the manuscript, our consideration in terms of data sources include the 12 databases and three websites.*

**2.** The study selection currently appears to read that if the PICO for your review is not clearly identifiable in the title / abstract that you will exclude the study. Within a review it is usual to retain ‘potentially eligible’ studies at this stage too, where aspects of your inclusion/ exclusion criteria for the review are not fully reported in the title / abstract of the retrieved citation but may be reported if the full article is read. The flow chart suggests that studies will be included if the abstract does not provide adequate detail – but the prose needs to reflect this too. eg as stated by Askey and O’Malley (2005) “If the relevance of a study is unclear from the abstract, then the full article will be obtained.”

***Response****: Sorry for the misunderstanding. In order to light more the reader, we added in the legend of figure 1 an explanation as follow: “The plain text twill be considered for reading”. This means that any inclusion at that stage remains potentially eligible and then will be reassessed in reading the plain text.*

**3.** Expert consultation section – states that informed consent will be obtained, but in the ethics declaration it states that consent is not required. Could the ethics statement be updated to better reflect that while consent is not required for the review, consent will be gained for the expert consultation workshops? This will provide better consistency through the manuscript.

***Response****:* *We thank you for this important comment. We formulate the following excerpt:* *Written informed consent will be required from the experts who will be joining the consultation session. Please, see on P 7.*

**4.** In the version provided Figure 1 does not states “ye” not “yes” and “exclu” not “exclude”. I presume this would be rectified if we had a copy of the actual Figure?

***Response****:* *Thanks. The wording has been adjusted in the algorithm in Figure 1.*

**5.** The definition of migrant no longer reads well – it would be better as "a person who has
voluntarily moved or is forced to move, whether within a country or across an international border, temporarily or permanently, and for a variety of reasons …”

***Response****: Thanks a lot for the suggestion. We truly found it very readable. So, we take it into account. Please see on P 4.*

**6.** I agree with the other reviewer that there is lack of clarity over the word ‘synthesize’. The authors need to give a plan for how data will be ‘synthesized’ within the expert consultation. Alternatively, would the word ‘summarize’ better describe the planned process that the word ‘synthesize’? This would also potentially more closely reflect the approach proposed by Askey and O’Malley (2005).

***Response****: Thank you. As suggested, we change the word ‘synthezise’ to ‘summarize’. Please see on P 7*
